# Effects of early and later timed cervical headgear treatment on the eruption timing and pattern of permanent upper canines and molars from 7 to 18 years of age: follow-up of a randomized controlled trial

**DOI:** 10.1093/ejo/cjaf056

**Published:** 2025-06-27

**Authors:** Katja Käsmä, Anna-Sofia Silvola, Ville Vuollo, Johanna Julku

**Affiliations:** Research Unit of Population Health, Faculty of Medicine, University of Oulu, Aapistie 5 B, 90220 Oulu, Finland; Research Unit of Population Health, Faculty of Medicine, University of Oulu, Aapistie 5 B, 90220 Oulu, Finland; Department of Oral and Maxillofacial Diseases, Oulu University Hospital, Kajaanintie 50, 90220 Oulu, Finland; Medical Research Center Oulu, Oulu University Hospital and University of Oulu, Aapistie 5 A, 90220 Oulu, Finland; Research Unit of Population Health, Faculty of Medicine, University of Oulu, Aapistie 5 B, 90220 Oulu, Finland; Department of Oral and Maxillofacial Diseases, Oulu University Hospital, Kajaanintie 50, 90220 Oulu, Finland; Research Unit of Population Health, Faculty of Medicine, University of Oulu, Aapistie 5 B, 90220 Oulu, Finland; Department of Oral and Maxillofacial Diseases, Oulu University Hospital, Kajaanintie 50, 90220 Oulu, Finland

**Keywords:** cervical headgear, canines, permanent molars, eruption, child, early treatment, orthodontics

## Abstract

**Background:**

Although cervical headgear (CH) is a widely studied method for the correction of Class II malocclusion, there is a lack of evidence regarding the effects of early versus later treatment timing on permanent upper canines and molars.

**Objectives:**

To evaluate the differences between early versus later cervical headgear treatment to the eruption time and inclination of maxillary permanent canines and second molars, inclination of the first molars, and the overlapping between second and third molars.

**Trial design:**

Randomized, parallel-group, prospective controlled trial on timepoints T0–T2, follow-up study on T2–T4.

**Methods:**

The material consisted of 67 seven-year-old children, with a Class II malocclusion, randomized into two equal-sized groups using opaque sealed-envelope randomization. In the early group (EG, n = 33), CH treatment was started at the age of 7.8 (T_0_) and in the later group (LG, n = 34) at the age of 9.5 (T_1_). Children received active CH treatment until Class I molar occlusion was achieved, after which individualized orthodontic treatment was provided at timepoints T2–T4. Dental panoramic tomographs (DPTs) were taken at five timepoints: T0–T4 (T0 = mean age 7.3 years, T1 = 9.6 years, T2 = 11.5 years, T3 = 15.3 years, and T4 = 17.8 years). The comparisons between the groups in DPT measurements were made at different timepoints using *t*-test and Mann–Whitney *U*-test and by combining the results using a linear mixed model. Due to the nature of the trial, the clinicians and children could not be blinded during the study; therefore, blinding was applied during data assessment.

**Results:**

In the LG, the right canine erupted earlier in T1 and in T2 and in combined results both canines erupted earlier. In the LG canines were more vertically inclined in T2. The second molars in the EG were more distally tipped in T1 and on the left side in combined results. The eruption stage of the second molars was earlier in the LG than in the EG. In the EG, more overlapping of the right and left third molars with the second molars was observed in T1. All P-values were < 0.05. No harms were encountered.

**Conclusion:**

A later treatment timing seems more beneficial, with earlier eruption and more vertically oriented canines, less distal tipping, and earlier eruption of second molars and less overlapping between second and third molars.

**Trial registration:**

Clinical Trials ID: NCT02010346

## Introduction

Cervical Headgear (CH) is a common appliance used primarily to correct Class II malocclusion and crowding. CH treatment is based on Kloehn’s work [1, 2]. Previous studies have shown its efficacy in achieving both skeletal and dental effects [3–5]. The main aims of CH treatment are to move the maxillary permanent teeth more distally [3, 6] and to restrain the growth of the maxilla [7, 8]. CH treatment also causes distal tipping of the maxillary permanent molars [9] especially when applying heavy forces [10]. The use of headgear treatment is effective for gaining space in dental arches [5] and treating moderate crowding [8]. Space is gained in both dental arches by widening and lengthening the arches [5, 8].

Previous studies in CH have shown it to influence the permanent canine location and inclination [3, 11, 12]. The median eruption age for maxillary canines in Finnish children is 10.8 years for girls and 11.3 years for boys [13]. The maxillary permanent canines are the second most frequent teeth to be impacted after the third molars [14]. The incidence of impaction of maxillary canine can vary racially and has been reported to vary from 0.92 per cent [14] to about 2 per cent [15, 16]. It has been shown that CH treatment influences the inclination of permanent canines, orienting them more vertically [11, 12]. The strongest influence was seen in right-side canines after 2 years of headgear treatment [12]. Changes in the vertical position of the canines are more pronounced in spaced dental arches than in crowded dental arches, suggesting a relationship between vertical orientation and space conditions of the dental arch [11].

However, the optimal timing of the treatment of Angle Class II malocclusion with CH is still somewhat controversial. When considering the timing of CH treatment, previous studies have reported variable outcomes. Kirjavainen reported that early CH treatment had a more pronounced effect on maxillary growth and widening [17]. Julku also found that early treatment was beneficial when considering the sagittal and transversal changes in the maxilla and mandible, especially among boys [4]. Additionally, the risk of relapse of the space gain seems to be smaller when CH is used as an early-phase treatment [5]. However, Tulloch stated that early two-phase treatment in mixed dentition, including CH and fixed appliances, may not be clinically more effective than one-phase treatment in the early permanent dentition [18]. Pirttiniemi [19] also noted that early CH treatment may result in a longer mean treatment time due to the two-phase treatment, potentially reducing patient compliance [20].

The median eruption age for maxillary second molars in Finnish children is 11.9 years for girls and 12.3 years for boys [13]. The second molars are shown to erupt more slowly with CH treatment; however, the second molars have fully erupted in later stages if nothing pathological is identified [21]. It has been suggested that the optimal timing for headgear treatment, when considering the second molars, is when radiographically the crowns of the second molars have erupted past the apical third of the roots of the first molars. This would prevent the impaction of the second molars [22]. If CH is used for an extended period, it may influence the second molars, causing a delayed eruption [23]. Therefore, our study aimed to investigate the early versus later CH treatment timing effects from the more specific perspective of permanent upper canines and molars, aspects that have not yet been thoroughly examined.

### Objectives

The aim of this study was to evaluate the effects of early and later timed Kloehn Type cervical headgear treatment on eruption time and pattern of upper permanent maxillary canines and second maxillary molars, the inclination of first permanent molars and overlapping between second and third molars.

## Materials and methods

### Trial design, ethical consideration and registration

This research is a randomized controlled trial from timepoints T0 to T2, and from T2 to T4 it serves as a follow-up study of the randomized clinical trial. The trial design was a prospective, parallel group, controlled, randomized (1:1 ratio) one designed according to the CONSORT guidelines. The study protocol was approved beforehand by the regional medical research ethics committee of the Wellbeing Services County of North Ostrobothnia, Finland (EETTMK: 46/2003) (14.10.2013) and by the health service authorities in the municipalities involved in the study. The trial is registered at ClinicalTrials.gov, number NCT02010346 (12/2013).

### Sample size calculation

The sample size was calculated with independent samples *t*-test with the power of 80% and a significance level of 0.05. Effect size estimates were based on previous study with a similar study design [8]. Effect size for ANB-angle was determined to be 2°C. According to the sample size calculation, an adequate power would be achieved with eleven subjects in each group. The intake of subjects was increased to secure the completion of the research because the study was longitudinal (the follow-up period being from ages 7 to 18) and because long studies are more prone to dropouts.

### Participants

**Figure 1.**
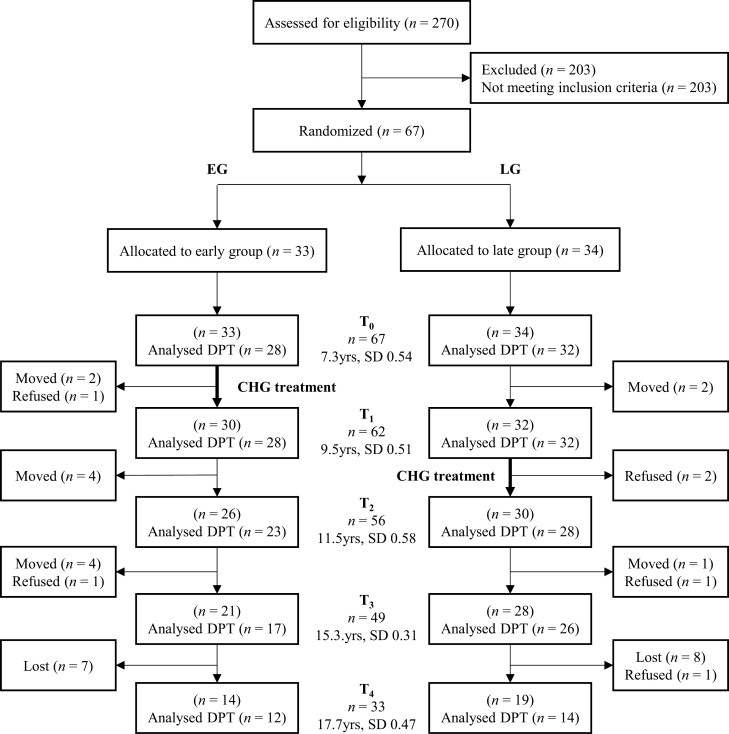
Chart illustrating the participant flow.

The participant flow during the study is illustrated in Fig. 1. A total of 270 healthy seven-year-old first graders were screened for the study in three Northern Finland municipalities between February 2004 and June 2008. Inclusion criteria for the study were Class II occlusion bilaterally and an overjet of > 6 mm or a deep bite. The exclusion criteria consisted of previous orthodontic treatment, inborn facial syndrome or severe facial asymmetry, suspected sleep-disorder breathing, chronic or recurrent upper airway infections and an angle over 35°C between the palatal line and the mandibular line (PL-LM angle).

The inclusion criteria for the study were met by 67 children (28 girls, 39 boys) with an average age of 7.2 years (SD = 0.55). All the children meeting the criteria agreed to participate in the study. The children and their parents were informed about the research, and they signed an informed consent. The children were randomly divided into two equal-sized groups: the early group (EG: n = 33, 13 girls, 20 boys) and the late group (LG: n = 34, 15 girls, 19 boys).

### Randomization

Randomization was performed using numbered, sealed, opaque envelopes prepared in advance, with each child selecting one envelope in a 1:1 allocation ratio. A note was given to the general dentist on which group and which treatment timing was assigned to the patient. The study groups and screening, randomization, sample size calculation and blinding have already been described in detail in earlier articles of this research [4, 24, 25]. Previous articles of this study have reported cast model and cephalometric analysis [4, 25, 26]. The follow-up period ended in 2018.

### Recruitment

Patient recruitment occurred from February 2004 to June 2008. DPT imaging was performed in five phases: initial scans (T0) from March 2004 to March 2008, follow up scans (T1) from June 2005 to November 2010, (T2) from February 2008 to June 2012, (T3) from February 2011 to December 2015 and (T4) from December 2013 to December 2017.

### Interventions

The cervical headgear therapy was performed by nine general dentists in three dental clinics of the public health services in Northern Finland. The treatment protocol for all the children was similar. The permanent first molars were fitted with molar bands with buccal tubes and a Kloehn-type CH with longer outer bows was used. The outer bows were bent upwards 10°C and the inner bows were expanded and held 5 mm wider than the distance between the tubes. A force of 500 g total, measured with orthodontic force gauge, was used and the children were instructed to wear the CH for 8–10 hours every night.

In the EG, the headgear therapy was carried out between timepoints T0 and T1 (onset at mean age 7.8 years, SD 0.48, range 6.8–8.5 years) and the LG in timepoints between T1 and T2 (onset at mean age 9.5 years, SD 0.59, range 8.8–11.2 years). Active CH treatment was carried out until Class I occlusion was achieved on the permanent first molar. In the EG, this took on average 1.6 years (SD 0.67) and in the LG 1.4 years (SD 0.80). After active treatment, CH was used as a retention with reduced wear for an average of 11 months in the EG and 14 months in the LG. No harmful effects were encountered during the study.

### Blinding

Blinding of patients, their parents, and the clinicians performing the orthodontic treatment was not possible due to the nature of the study. However, blinding was applied during outcome assessment. Before the study began, each patient was assigned a code, and all collected material was coded accordingly, ensuring that data collectors, outcome adjudicators, and data analysts were unable to identify the patients or their group assignments.

### Outcomes

Dental panoramic tomographs (DPTs) were taken during the study at in five timepoints T0–T4 (T0 = mean age 7.3 yrs (SD 0.55), T1 = 9.6 yrs (SD 0.51), T2 = 11.5 yrs (SD 0.58), T3 = 15.3 yrs (SD 0.40), T4 = 17.8 yrs (SD 0.35)). One subject had no DPT images taken, and five subjects had only 1 DPT taken. Hence, they were excluded from the study, because it would have been impossible to compare potential changes. Some subjects dropped out of the study or had missed imaging, the final study material consisting of 61 subjects and a total of 240 DPT images.

**Figure 2.**
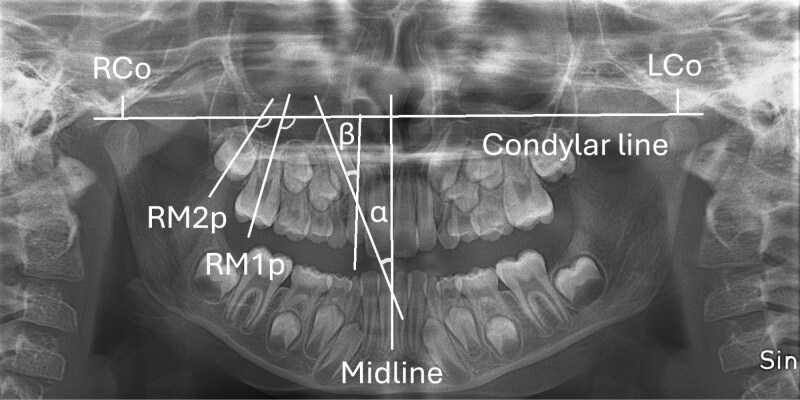
Illustration of the angle measurements of canines and first and second maxillary molars from the DPT. The canine angulation was assessed by measuring the angle (α) between the mid-axis of the canine and the maxillary midline and the angle (β) between the mid-axis of the canine and the mid-axis of the lateral incisor. Landmarks were used in the DPT analysis of the first and second molars’ inclinations. RCo: the most superior point of the right condyle. LCo: the most superior point of the left condyle. Condylar line: the line between RCo and LCo. RM2p: perpendicular line of the right second molar mid-axis. RM1p: perpendicular line of the right first molar mid-axis. Molar inclinations were assessed with the angle between the condylar line and the perpendicular line of the mid-axis of the molar crown.

**Figure 3.**
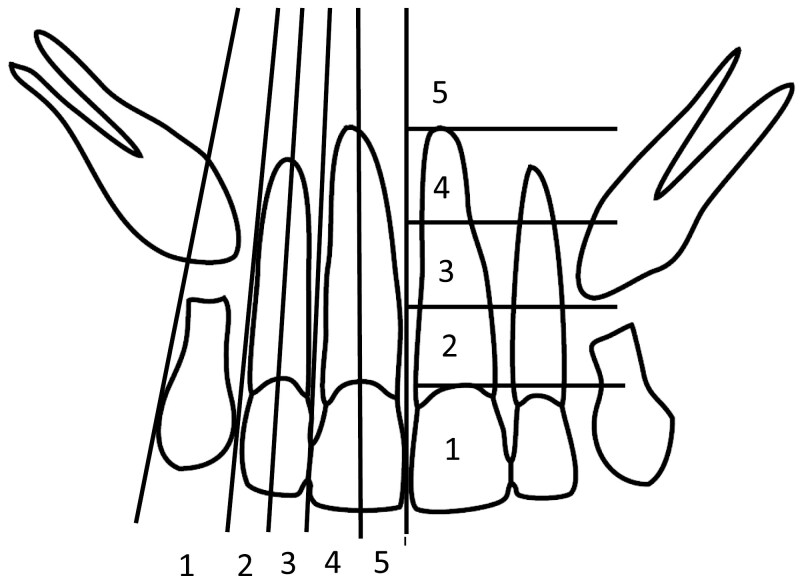
The horizontal sector of the canine was classified by comparison to the incisors. (1) No canine overlapping occurs with the incisors, the canine is overlapping (2) distal half of the lateral incisor, (3) mesial half of the lateral incisor, (4) distal half of the lateral incisor, (5) mesial half of the mesial incisor. The vertical position of the canine was assessed by comparing the location of the occlusal tip of the canine to the mesial incisor. (1) Tip of the canine above the cementoenamel junction of the mesial incisor, (2) at the level of the cervical third of the root, (3) at the level of the middle third of the root, (4) at the level of the apical third of the root, (5) underneath the apex of the mesial incisor.

**Figure 4.**
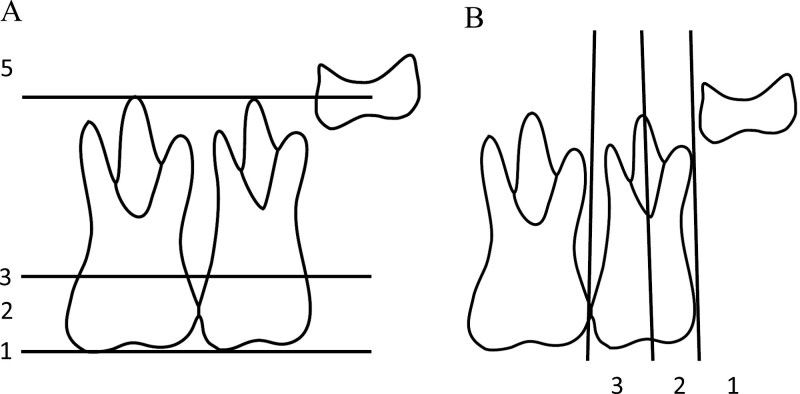
A) The classification of the second molar eruption was assessed by comparing it to the first molar. (1) The occlusal surface of the second molar at the same level as the occlusal surface of the first molar, (2) the occlusal surface above the cementoenamel junction, (3) the occlusal surface at the same level as the cementoenamel junction, (4) the occlusal surface beneath the cementoenamel junction, (5) the occlusal surface above the apex of the first molar roots. B) Classification of the overlapping of the third molar compared to the second molar. (1) Third molar not overlapping the second molar, (2) third molar overlapping the distal half of the second molar, (3) third molar overlapping the mesial half of the second molar.

The measurements for the DPTs were made using the ImageJ software (release 1.53n; National Institutes of Health, Wayne Rasband and contributors, USA) The angle of the canine was measured using the Ericson and Kurol method [27]. Angle α was measured between the mid-axis of the maxillary canine and maxillary midline, and angle β was measured between the mid-axis of the maxillary canine and the mid-axis of the lateral incisor (Fig. 2). The angles of the maxillary first and second molars were measured using a modified version of the Talvitie method (Fig. 2) [10]. The measurements were made between the condyle line and the mid-axis of the first and second molars. The vertical position of the canine was determined using the Power and Short method (Fig. 3) [28], but with the comparison made to the central incisor instead of the lateral incisor, because the lateral incisor had not fully erupted in the early DPTs. The overlapping of the canines with the incisors was assessed using the Ericson and Kurol method (Fig. 3) [27]. The development stage of the second molar was assessed using a modified version of the Demirjian method for the development stages of the second molar [29]. The eruption stage of the second molar was assessed by comparing it to the first molar (Fig. 4A). The overlapping between the second and third molars was also assessed (Fig. 4B). All measurements were made bilaterally.

The maxillary second molar development was assessed using a modification of the eight-level classification by Demirjian: (1) the beginning of the cusp tip calcification is visible, (2) the mineralized cusp tips have fusioned, (3) the dentine has started forming and the crown is halfway formed, (4) the crown is formed to the cementoenamel junction, and the beginning of root formation is visible, (5) the root length is less than the crown height and the initial formation of the bifurcation area is seen to be calcificating, (6) the root length is equal or longer than the crown height and the roots have funnel-shaped endings, (7) the root walls are parallel with the apex still open, (8) the tooth formation is completed and the apex is closed.

### Statistical analysis

Statistical analyses were carried out with R software environment version 4.3.1. The radiographic analysis was performed by the same investigator. To determine the method error, 20 randomly selected DPTs were measured twice, and intra-rater reliability was measured with the intra-class correlation coefficient (ICC). The ICCs for all measurements were excellent (range 0.9–1). The effect of treatment timing on the measurements was analysed using linear mixed models (LMM). LMM considers dependence within patients in different time points by using random intercepts. Age at timepoint and gender were set as confounding variables. Interaction between age and group was also tested, but it did not improve the model based on the Akaike information criterion. Group measurements were also compared separately in each time point. Normally distributed dependent variables were tested using Student’s *t*-test, while Mann-Whitney *U* test was used for skewed and ordinal data. Normality was tested using the Shapiro-Wilk test. A threshold of 0.05 was chosen as the level of significance for *P-*value.

## Results

**Figure 5.**
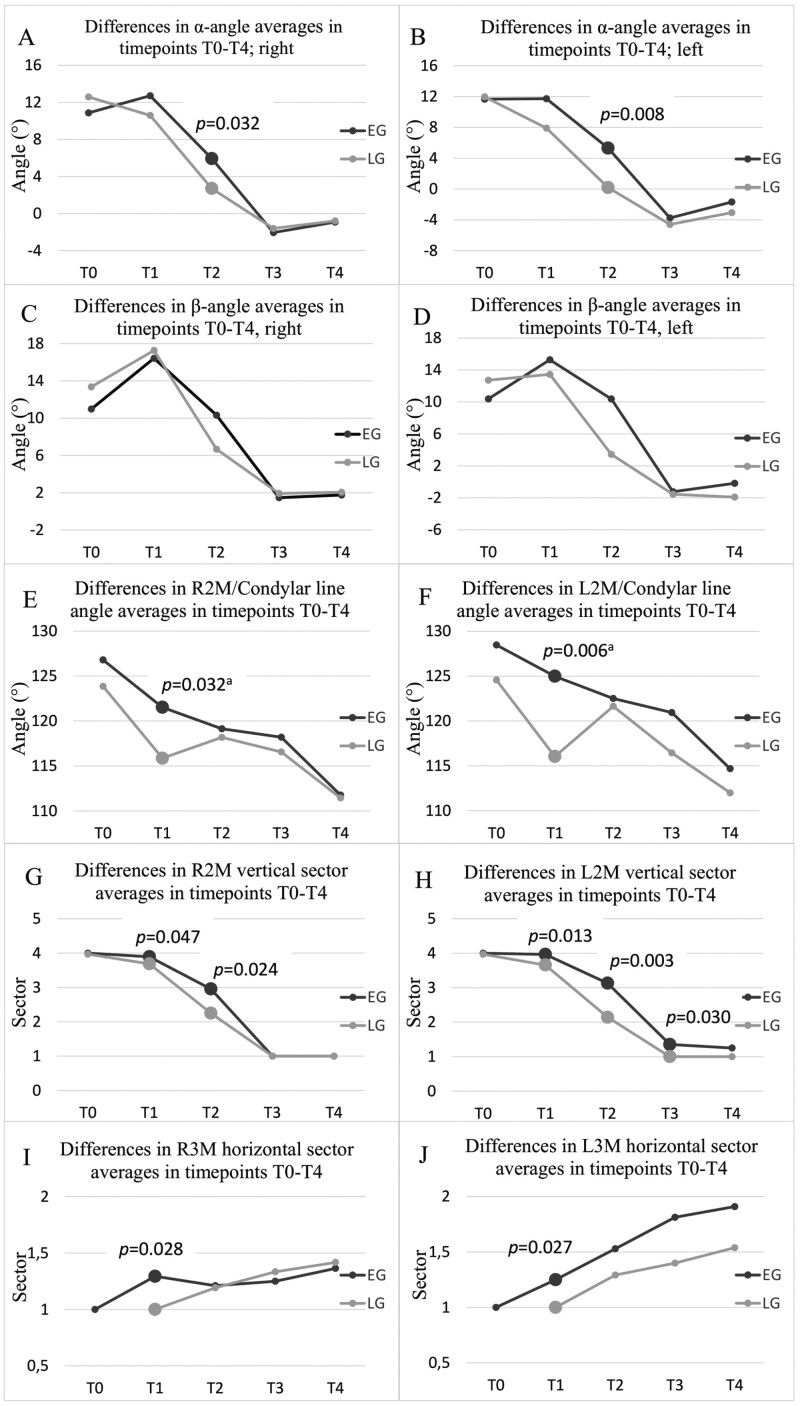
Illustrations of the differences between the EG and the LG in timepoints T0-T4. Results with significant differences are marked using larger dots and *P*-values are reported. The statistical analysis was made using Mann-Whitney *U* test and Student’s *t*-test^a^. (A) Differences in right canine α-angle. (B) Differences in left canine α-angle. (C) Differences in right canine β-angle. (D) Differences in left canine β-angle. (E) Differences in R2M/condylar line angle averages. (F) Differences in L2M/condylar line angle averages. (G) Differences in R2M eruption stage averages. (H) Differences in L2M eruption stage averages. (I) Differences in R3M/R2M overlapping averages. (J) Differences in L3M/L2M overlapping averages.

Fig. 5 shows the differences between the groups in different timepoints in inclination of the canines (A, B, C, D), in second molar inclination and eruption stage (E, F, G, H), and overlapping between second and third molar (I, J). There was a significant difference in α-angle in T2 on the right and left canine which were more vertical in the LG that in the EG (*P* = 0.032, *P* = 0.008, respectively) (Fig. 5A, B). At the end of the follow-up period, the results showed no significant difference between the groups in canine inclination. No significant differences were found in canine overlapping between the groups.

The combined results of different timepoints of the upper canines’ eruption, upper second molars’ angulation compared to the condylar line and the second molars’ eruption are shown in Table 1. The treatment timing had a statistically significant effect on the right and left canine vertical location (*P *= 0.012, B = −0.25, *P* = 0.038, B = −0.21, respectively) and on the and left second molar angulation compared to the condylar line *(P *= 0.041, B = −3.35), and right and left second molar eruption (*P* = 0.004, B = −0.24, *P* = 0.000, B = −0.42, respectively). The LG had their canines more erupted in earlier stages compared to the EG. When comparing the groups in different timepoints, there was a significant difference in T1 and in T2 on the right canine vertical sector, with the LG having their canines more erupted (*P* = 0.042, *P* = 0.031, respectively).

**Table 1.** Linear mixed models for the right and left canines’ eruption, the angle between right (R2Mp) and left (L2Mp) second molars’ perpendicular line between the condylar line and eruption of right and left second molars.

	Right canine eruption					Left canine eruption		
	B	CI 95%		P	B	CI 95%		P
		Lower	Upper			Lower	Upper	
**Intercept**	5.08	4.74	5.43	** < 0.001**	4.93	4.58	5.28	** < 0.001**
**Group (Late)**	−0.25	−0.44	−0.06	**0.012**	−0.21	−0.40	−0.01	**0.038**
**Age**	−0.26	−0.28	−0.23	** < 0.001**	−0.25	−0.28	−0.23	** < 0.001**
**Sex (Male)**	−0.05	−0.24	0.15	0.622	0.04	−0.16	0.23	0.712

	R2Mp/condylar line angle				L2Mp/condylar line angle			
	B	CI 95%		P	B	CI 95%		P
		Lower	Upper			Lower	Upper	
**Intercept**	133.45 −2.70 −1.01 −1.90	129.09	137.80	** < 0.001**	135.44	131.28	139.54	** < 0.001**
**Group (Late)**		−5.80	0.39	0.086	−3.35	−6.56	−0.15	**0.041**
**Age**		−1.30	−0.72	** < 0.001**	−0.95	−1.21	−0.70	** < 0.001**
**Sex (Male)**		−5.05	1.24	0.231	−2.62	−5.88	0.65	0.114

		R2M Eruption			L2M Eruption			
	B	CI 95%		P	B	CI 95%		P
		Lower	Upper			Lower	Upper	
**Intercept**	6.78	6.49	7.08	** < 0.001**	6.76	6.44	7.08	** < 0.001**
**Group (Late)**	−0.24	−0.41	−0.08	**0.004**	−0.42	−0.61	−0.23	** < 0.001**
**Age**	−0.34	−0.36	−0.32	** < 0.001**	−0.33	−0.35	−0.30	** < 0.001**
**Sex (Male)**	−0.04	−0.21	0.12	0.605	−0.03	−0.22	0.16	0.77

B = coefficient; CI = confidence interval.

Considering the tilting of the first maxillary molars, statistically significant difference was found at T1 on the left side, with the EG having more distally tilted first maxillary molar (*P *= 0.047). When comparing the tilting of the right (R2M) and left (L2M) second maxillary molar in different timepoints (Fig. 5E, F), significant differences were seen in T1. At T1, the second molars were more distally inclined in the EG than in the LG (R2M; *P* = 0.032, L2M; *P* = 0.006, respectively). Also, a significant difference was found in the combined results in the angulation of the L2M compared to the condylar line (*P *= 0.041, B = −3.35) (Table 1), with the EG having their left second molar more distally tipped.

The results (Fig. 5G, H) also indicated that the second molars were more erupted at earlier stages in the LG compared to the EG in T1, T2 on the right side (*P* = 0.047, *P* = 0.024, respectively) and in T1, T2 and T3 on the left side (*P* = 0.013, *P* = 0.003, *P* = 0.030, respectively). The combined results also indicated that the second molars erupted earlier in the LG (R2M; *P* = 0.004, B = −0.24, L2M; *P* = 0.000, B = −0.42) (Table 1). In the EG, more overlapping of the third molar with the second molar was observed in T1 on both sides (R3M; *P* = 0.028, L3M; *P* = 0.027) (Fig. 5I, J). However, the differences evened out in later stages and there were no significant differences in later timepoints.

## Discussion

The aim of this study was to investigate the effects of early and later timed cervical headgear treatment and its effects on permanent upper canines and first, second and third permanent molars. The results of this study indicate that, when considering the timing of the CH treatment, a later treatment time may be more beneficial for dental effects on upper permanent canines and molars.

The results indicated that a later treatment timing would be beneficial in terms of the eruption timing and inclination of the permanent canines. Comparing the two groups in different timepoints showed that the right canine erupted earlier in timepoints T1 and T2 in the LG than in the EG, and the same conclusion was drawn with both right and left canines from the combined results. Previous studies have shown that headgear influences the canine vertical orientation, orienting them more vertically [11, 12], and this study showed that the inclination of the canine in the LG was more vertically oriented than the EG in T2. These differences could be explained by the later treatment timing being closer to the normal eruption age of the canines, which in Finnish children is 10.8 years for girls and 11.3 years for boys [13], and therefore canines are more affected by the treatment. In this study, no primary problems existed in canine eruption, but these results may have clinical significance when treating mesially inclined canines. CH has been shown to widen the upper arch [4, 5, 25, 30] and it has also been shown that widening of the upper arch at the age of 9 years has a positive effect on maxillary canine eruption [31]. Based on these results, it can be assumed that gaining space through widening and/or lengthening of the dental arch may facilitate the eruption of the canine. No differences in the overlapping of canines with incisors were detected between the groups.

Worms [32] stated that the resistance to tooth movement comes from the periodontal membrane, the alveolus and adjacent contacting dental units. Authors also stated that unerupted second molars less frequently cause resistance for first molar distalisation since they are not in contact with first molars. However, the second molars and the third molars, through the movement of second molars apices, move more distally along the first molars. Kinzinger [33] showed that in the direction of distalisation, a toot bud acts like a fulcrum on the mesial neighbouring tooth. In case of the first molar, the eruption stages of the second molar had an impact on the distalisation of the first molars. More tipping of the first molars occurred in patients with their second molars still at the budding stage. Also, more tipping of the second molars were observed when they had fully erupted with the third molars at budding stage in the direction of movement.

The study showed results similar to earlier studies [9, 10, 34], with CH causing distal tipping of the permanent second molars. When considering the inclination of the second molars, our study showed that a later treatment plan seems to be more beneficial. Early treatment timing caused more distal tipping of the second molars. Thus, in case of distally inclined second molars, early treatment could aggravate distal inclination. The results at different time points showed that the difference of the distal tipping of the second molar is most pronounced in T1 when the LG had not yet received treatment. These differences, however, even out in later stages and no significant differences are seen in later timepoints. Different treatment timing had a significant effect on first molar inclination on the left side with the EG having more distally tipped first molar. Thus, the effect was seen only on the left side and the significance level was near to non-significance level the clinical value of this observation is limited. The result could be stronger in a larger sample, but since both groups had fully erupted first molars at the start of the treatment the difference between groups may be insignificant.

The results advocated for later treatment, also when considering the eruption of the second molars. The eruption stage of the second molars was slower in the EG compared to the LG in T1 and T2, and in T3 on the left side. Moreover, the combined results showed later treatment to cause the molars to erupt earlier. The results of the impact of CH on the third molar may support later treatment since less overlapping between the second and third molars was seen in the LG. However, the difference in the overlapping of the second and third molars between the groups was seen only at age 9.5 and levelled off in later stages. Thus, the clinical significance of the results cannot be confirmed.

### Strengths and limitations

One strength of this study is that the beginning of the study is randomized and blinded, and the follow-up period is long. One limitation of this study is that the patients received additional orthodontic treatment during the study period, but since the participants met the national criteria for orthodontic treatment, it would have been ethically wrong to leave the patients untreated. However, in the early phases of the treatment most of the patients had only CH. Additional orthodontic treatment, such as treatment with fixed appliances, was implemented in later stages of the treatment. Thus, the effect of the additional orthodontic treatment had only a minor effect on the inclination of the canines and molars and the most significant differences in this study were seen in T1 and T2.

## Conclusion

According to this study, a later treatment plan seems to be more beneficial when considering the dental effects of CH on the permanent upper canines and molars. The upper canines in the LG erupted earlier and were more vertically oriented. Later CH treatment also seemed to be more beneficial for better vertical inclination and for earlier eruption of the upper second molars, along with reduced overlap between the second and third molars. No differences were found between the two groups in the overlapping of canines and incisors.

## Data availibility

Data underlying this publication will be shared on reasonable request to the corresponding author.
